# Decreased functional connectivity and structural deficit in alertness network with right-sided temporal lobe epilepsy

**DOI:** 10.1097/MD.0000000000010134

**Published:** 2018-04-06

**Authors:** Yujun Gao, Jinou Zheng, Yaping Li, Danni Guo, Mingli Wang, Xiangxiang Cui, Wei Ye

**Affiliations:** a Department of Neurology; b Department of Radiology, the First Affiliated Hospital of Guangxi Medical University, Nanning, Guangxi, China.

**Keywords:** alerting network, attentional network test, diffusion tensor imaging, resting-state functional magnetic resonance, temporal lobe epilepsy

## Abstract

Patients with temporal lobe epilepsy (TLE) often suffer from alertness alterations. However, specific regions connected with alertness remain controversial, and whether these regions have structural impairment is also elusive. This study aimed to investigate the characteristics and neural mechanisms underlying the functions and structures of alertness network in patients with right-sided temporal lobe epilepsy (rTLE) by performing the attentional network test (ANT), resting-state functional magnetic resonance imaging (R-SfMRI), and diffusion tensor imaging (DTI).

A total of 47 patients with rTLE and 34 healthy controls underwent ANT, R-SfMRI, and DTI scan. The seed-based functional connectivity (FC) method and deterministic tractography were used to analyze the data.

Patients with rTLE had longer reaction times in the no-cue and double-cue conditions. However, no differences were noted in the alertness effect between the 2 groups. The patient group had lower FC compared with the control group in the right inferior parietal lobe (IPL), amygdala, and insula. Structural deficits were found in the right parahippocampal gyrus, superior temporal pole, insula, and amygdala in the patient group compared with the control group. Also significantly negative correlations were observed between abnormal fractional anisotropy (between the right insula and the superior temporal pole) and illness duration in the patients with rTLE.

The findings of this study suggested abnormal intrinsic and phasic alertness, decreased FC, and structural deficits within the alerting network in the rTLE. This study provided new insights into the mechanisms of alertness alterations in rTLE.

## 1 Introduction

Patients with temporal lobe epilepsy (TLE) usually exhibit broad cognitive deficits,^[1,2]^ especially memory disorders,^[3–5]^ lower intelligence,^[6]^ and attentional dysfunction.^[2]^ The attentional network comprises 3 components: alerting, orienting, and executive.^[7,8]^ Alerting is defined as achieving and maintaining a state of high sensitivity to incoming stimuli. Increasing evidence has demonstrated that alerting functional abnormalities were unlikely interpreted by disturbances of a single brain region; however, several brain regions have been postulated to be crucial in alerting network.^[9,10]^ For example, Sturm et al^[9]^ found that the anterior cingulate gyrus, thalamus, middle temporal gyrus, and so on, participated in alertness function. The resting-state functional magnetic resonance (R-SfMRI) studies demonstrated that the inferior parietal and prefrontal cortices and thalamus were commonly involved in alertness function.^[11,12]^ Further, the intraparietal sulcus, anterior cingulate, anterior insular cortices, and thalamus were reported to be activated by an alerting task.^[13]^

The aforementioned studies elucidated that many brain regions participate in alerting function. However, data on some regions were still inconsistent between studies. This inconsistency may result from extremely small sample sizes and different methods used in different studies. Another possibility is that the alerting network is dynamic, and it exhibits interactions between multiple brain regions. The function and structure are inseparable. A few studies assessed the brain white matter tract regions associated with alerting. Certainly, expanding the sample size before studying the structure is particularly significant to determine better the effect that different brain structures and networks have on alerting.

Many studies have suggested that thalamus is associated with alerting function.^[9–12,14,15]^ In the present study, the right thalamus was selected as a seed, and a seed-based functional connectivity (FC) was performed to determine the brain regions involved in the alerting network. The seed-based FC is a commonly used method for analyzing the R-SfMRI data that are involved in calculating the correlations between the seed and other brain regions. R-SfMRI studies reported that FC was measured in schizophrenia,^[16,17]^ Alzheimer's disease,^[18]^ Parkinson's disease,^[19,20]^ and major depressive disorder.^[21]^ The seed-based FC method was also employed to determine the brain regions related to the default-mode network (DMN).^[22,23]^ Nevertheless, a few researchers studied the alerting network in rTLE.

Diffusion tensor imaging (DTI) and deterministic fiber tractography are proven techniques for deriving quantitative measurements of white matter fiber connection and microstructure. These techniques are employed extensively in various studies starting from healthy groups with different ages to patient groups with late-onset depression,^[24,25]^ Alzheimer's disease,^[26]^ and stroke.^[27]^ Alexopoulos et al^[28]^ even found that DTI could be used to investigate the relationship between white matter integrity and treatment effect. Nonetheless, the aforementioned method has not been applied in alertness-related brain regions of patients with rTLE. Hence, employing the approach to exploit white matter alterations of alertness-related brain regions to determine the seed-based FC is important. Recently, at least 2 studies, using R-SfMRI in conjunction with DTI-based tractography, reported white matter changes in the DMN.^[29,30]^ Therefore, this study hypothesized that patients with rTLE would show white matter deficits of connecting brain regions associated with alertness function. Moreover, epilepsy is a repeated discharge process, and brain damage and cognitive impairment may be aggravated by repeated damage. Hence, this study also assumed that some correlations existed between regional white matter alterations in fractional anisotropy (FA) of alerting network and clinical characteristics.

## 2 Materials and methods

### 2.1 Subjects

A total of 47 patients with rTLE and 34 healthy controls, recruited from the Epilepsy Clinic of the Department of Neurology of the First Affiliated Hospital of Guangxi Medical University, were included in this study. They were all right handed. The control group was matched for age, sex, and educational level with the patient group. The diagnosis of rTLE was based on the diagnostic manual from the International League Against Epilepsy.^[31]^ Patients with epilepsy who met any 2 of the following symptoms were classified as patients with rTLE: the clinical onset of symptoms suggested epileptic foci in the temporal lobe, such as psychiatric symptoms, abnormal emotional experiences, automatisms, epigastric rising, or dystonic posturing of the limb; the imaging results showed sclerosis or atrophy in the right hippocampus; and the interictal electroencephalographic (EEG) traces suggested abnormality in the right temporal lobe. The exclusion criteria for all subjects included the following: left handed, structural MRI finding identifiable focal abnormalities other than hippocampal sclerosis atrophy, history of serious medical diseases or other neurological illness, any lifetime psychiatric disorder, and score less than 24 in the Mini-Mental State Examination (MMSE). Written informed consent was obtained from all participants prior to the study. The study was approved by the ethical committee of the First Affiliated Hospital of Guangxi Medical University.

### 2.2 Behavioral paradigms

The ANT was devised by Fan and his colleagues.^[32]^ In the center of the screen a “+” was displayed as the fixation point. The stimulus signals could appear above or below the center of the screen in the form of a target (“→”) or a foil (“∗”). The foil could appear in one of the following 4 conditions: no foil, one foil at the center, one foil above and another one below the center, and one foil either above or below the center. The target could appear in one of the following 3 conditions: a single arrow, 5 arrows in the same direction, and 5 arrows in different directions. The subjects were required to correctly and quickly determine the orientation of the target. The alertness network RT was calculated by subtracting the double-cue RT from the no-cue baseline RT.

### 2.3 Scan acquisition

All participants were scanned on a 3T Achieva MRI scanner (Philips, The Netherlands). They were instructed to lie still and stay awake with their eyes closed. A prototype quadrature birdcage head coil fitted with a foam padding was applied to limit the head motion. The scanning parameters were as follows: structural scan (T1-weighted): spin-echo sequence, repetition time (TR) = 20 ms, echo time (TE) = 3.5 ms, slice thickness = 1 mm, and field of view (FOV) = 24 × 24 cm^2^, and scan time about 7 minutes. DTI scan: repetition time/echo time (TR/TE) = 8236/68 ms, slice thickness = 2 mm, pitch = 0 mm, and FOV = 224 × 224 mm^2^. The diffusion-sensitizing gradients were applied along the 32 noncollinear directions (*b* = 1000 m^2^/s) with 2 acquisitions without diffusion weighting (*b* = 0), and scan time about 10 minutes. R-SfMRI scan: gradient echo–echoplanar imaging sequence (echoplanar imaging T2∗ weighted), TR/TE = 2000/30 ms, slice thickness = 5 mm, pitch = 1 mm, FOV = 220 × 220 mm^2^, and flip angle = 90°, and scan time about 8 minutes.

### 2.4 Data preprocessing

Imaging data of R-SfMRI were preprocessed using the RESTplus software (http://www.restfmri.net/) in MATLAB (Mathworks). The first 5 time points were removed. First, the slice timing and head motion were corrected. No participants had more than 2 mm of maximal translation and more than 2° of maximal rotation. Then, the images were segmented into T_1_ images, which were subsequently normalized in the standard Montreal Neurological Institute (MNI) echoplanar imaging space, and resampled with 3 × 3 × 3 mm^3^ resolution. The obtained images were subsequently smoothed with a Gaussian kernel of 8-mm full-width at half-maximum, and band-pass filtered within 0.08 to 0.1 Hz, following a linear detrend. Several spurious covariates were removed, including 6 head-motion parameters obtained by rigid body correction, signal from a ventricular region of interest (ROI), and signal from a region centered in the white matter. The global signal was not regressed out because it was saved in the processing of FC data.^[33]^

The imaging data of DTI were preprocessed using the PANDA software^[34]^ in MATLAB (Mathworks). The DICOM files were converted into NIFTI images by extracting the brain tissue, cutting off the nonbrain space in the raw images, and cropping the gap between the border of an image and the border of the brain. The head motion and eddy-current distortions were corrected using an affine transformation of each diffusion-weighted image to the nondiffusion-weighted (*b* = 0) image as reference volume. The brain mask was estimated using the bet command of FMRIB Software Library. Thus, images of FA were generated for each subject by applying an FMRIB58_FA template. Individual FA images were registered to the FA template in the MNI standard space. From these new images, a mean FA image was created and thinned to create a mean FA skeleton using an FA threshold value of 0.2. Finally, the aligned FA data of each subject were projected onto the skeleton by assigning each voxel to the maximum FA value detected in a direction surrounding the tract.

### 2.5 Seed-based FC processing

The right thalamus from the *Anatomical Automatic Labeling* (AAL) template was selected as a seed for global brain FC processing using RESTplus. For each participant, the seed-based FC was computed as Pearson correlation coefficients between the seed and other voxels of the whole brain. The correlation coefficients were then *z*-transformed for the standard purpose, and seed-based FC maps were generated. For this seed and each group, the seed-based FC maps were analyzed with one-sample *t* tests to detect voxels exhibiting a significant correlation with the seed. The significance level for each group was set at *P* < .01 corrected for multiple comparisons using the Gaussian Random Field (GRF) theory (voxel significance: *P* < .001, cluster significance: *P* < .01).

### 2.6 Deterministic tractography

The decreased FC regions (right parahippocampal gyrus, right superior temporal pole, right lingual gyrus, right insula, right putamen, right caudate, right superior temporal gyrus, and right amygdala) were extracted as ROI using PANDA. Subsequently, deterministic tractography was performed using PANDA to gain FA, fiber number (FN), and fiber length (FL) of these regions.

### 2.7 Statistical analyses

Group differences were compared using voxel-wise two-sample *t* tests. Age, sex, and years of education were used as covariates in group comparisons to reduce the possible effects of these variables. Since FC results might be affected by head micromovement from volume to volume, the frame-wise displacement values were computed and also used as a covariate in group comparisons. The significance level for each group was set at *P* < .01 (GRF corrected). The group comparisons were restricted to voxels, with significant correlation FC maps of the 2 groups using a mask from the union set of one-sample *t* test results. Voxel-based correlations were conducted between abnormal FC/FA values of each patient and clinical variables using the IBM SPSS Statistics 22.0 software.

## 3 Results

### 3.1 Behavioral results and clinical characteristics of subjects

The demographic and clinical characteristics of participants are shown in Table 1. The 2 groups exhibited no significant differences in age, sex ratio, years of education, MMSE, and alertness network RT, but had longer intrinsic and phasic alertness RT.

**Table 1:**
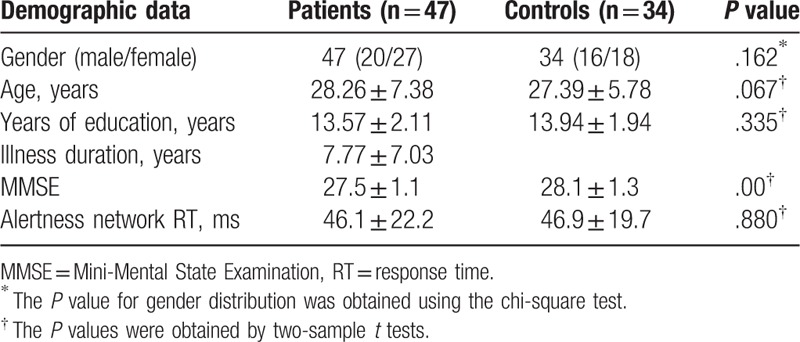
Characteristics of the participants.

### 3.2 Seed-based FC analyses

**Figure 1:**
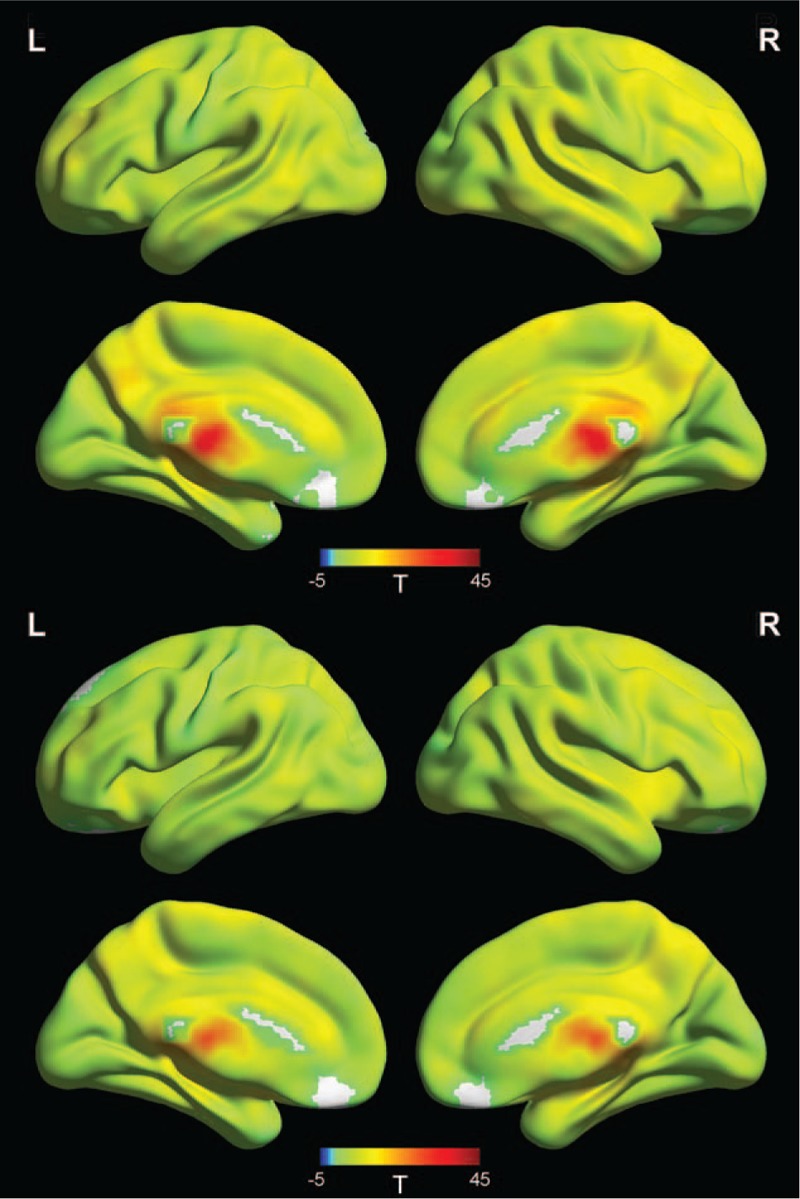
Mean FC strength maps within the patient and control groups. One-sample *t* tests on individual FC values were conducted at each voxel. Correction for multiple comparisons was conducted based on the GRF theory (voxel significance: *P* < .001, cluster significance: *P* < .01). FC = functional connectivity.

The one-sample *t* test results revealed that the right thalamus had positive FC with right superior frontal cortex, right precuneus, right middle temporal gyrus, right middle frontal gyrus, and right inferior temporal gyrus in the control group, and right superior frontal cortex, right precuneus, right middle temporal gyrus, right middle frontal gyrus, and right inferior temporal gyrus in the patient group (Fig. 1).

### 3.3 Seed-based FC analyses: group comparisons

**Figure 2:**
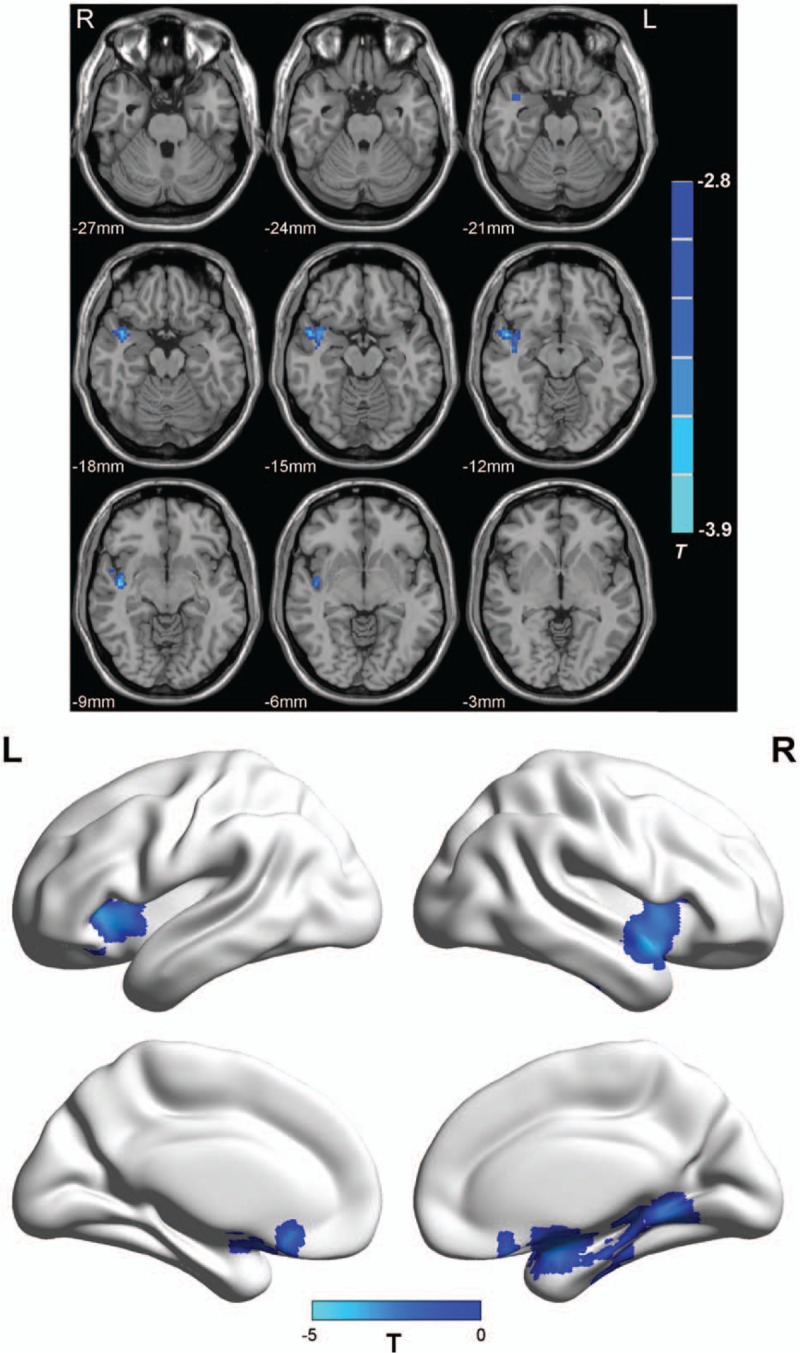
Statistical maps showing seed-based FC differences between the subject groups. Blue denotes lower FC values in the patients, and the color bar indicates the *t* values from two-sample *t* tests. Correction for multiple comparisons was conducted based on the GRF theory (voxel significance: *P* < .001, cluster significance: *P < .*05). FC = functional connectivity, GRF = Gaussian random field.

As shown in Figure 2, the patients exhibited significantly decreased FC but had longer intrinsic and phasic alertness RT compared with the controls. No significant increase in FC was found in the patients with rTLE.

### 3.4 FA, FN, and FL values between groups

FA significantly decreased in the right insula, parahippocampal gyrus, amygdala, superior temporal pole, and superior temporal gyrus. FN significantly decreased in the right superior temporal gyrus and right superior temporal pole. FL significantly decreased in the right parahippocampal gyrus and right amygdala in the patient group than in the control group (Table 2).

**Table 2:**
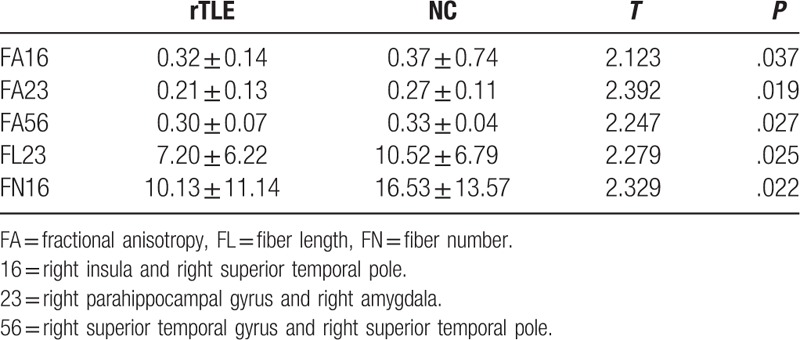
Significant differences in the FA, FL, and FN values between groups.

### 3.5 Correlations between abnormal *z*FC/FA and clinical variables in the patients

**Figure 3:**
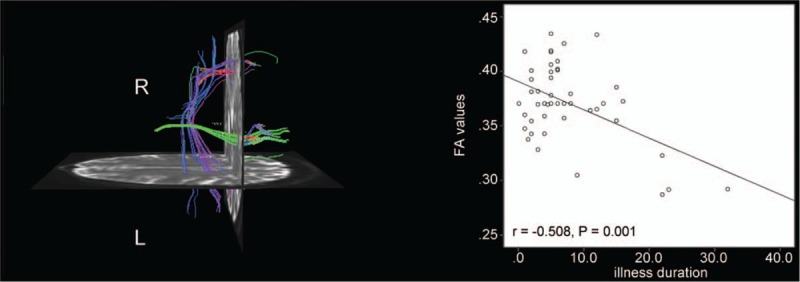
Abnormal tracks between right insula and right superior temporal pole and significantly negative correlations between abnormal FA values (right insula and right superior temporal pole) and illness duration in the patients with rTLE. FA = fractional anisotropy, TLE = temporal lobe epilepsy.

The voxel-based correlation method demonstrated that no significant correlations existed between the *z* values of the decreased region connectivity and the illness duration (*r* = 0.264, *P = .*073 > 0.05), but significant negative correlations existed between FA (right insula and right superior temporal pole) and illness duration (*r* = −0.508, *P = .*001) in the patient group (Fig. 3). No other correlations were observed in the patient group.

## 4 Discussion

The right thalamus from AAL was selected as a seed to examine the whole-brain FC in the present study. The right thalamus exhibited decreased FC in the brain regions within the right IPL, amygdala, and insula in the patients with rTLE than in the controls. No correlation was found between the abnormal zFC strength and clinical variables. Furthermore, deterministic tractography was employed to track the aforementioned regions, and the FA values in the white matter of right insula, parahippocampal gyrus, amygdala, superior temporal pole, and superior temporal gyrus of the patients with rTLE were found to be lower than those of the healthy controls. Moreover, a significantly negative correlation was found between abnormal FA (between the right insula and superior temporal pole) and illness duration in the patients. Behaviorally, longer intrinsic and phasic alertness RTs were detected. However, no significant differences were observed in the alertness network RT in the patients.

The ANT was widely employed to assess the alerting function in autism,^[35]^ multiple sclerosis,^[36]^ Alzheimer's disease,^[37]^ and healthy participants.^[14]^ The results of the present study indicated deficits in intrinsic and phasic alertness, but no significant differences were found in the alertness effect between the patients with rTLE and controls. The possible reasons for this finding were as follows: First, patients with rTLE were worse than normal in intrinsic and phasic alertness, but the alertness signal could help increase the sensitivity of alertness. Second, the time of changing clues was faster, and the participants were susceptible to distraction.^[38,39]^

Many studies suggested that the regions with alerting function were dorsolateral prefrontal cortex,^[11,13]^ frontal eye fields,^[13]^ anterior cingulate,^[9,11,13]^ temporal lobe,^[9,12]^ anterior insular cortices,^[13]^ thalamus,^[9,13,40,41]^ right putamen,^[42]^ and caudate nucleus.^[35]^ Howes and Boller^[43]^ found that alertness was impaired after lesions in the right hemisphere. Posner and Petersen^[8]^ proposed a right hemisphere noradrenergic alerting network including locus coeruleus, frontal areas, parietal cortex, and thalamus. A previous study also found that the effects of TLE on the brain were asymmetric.^[44]^ Dupont et al^[45]^ found decreased activation in the right frontal temporal and parietal cortex. Sturm et al^[9]^ exhibited that intrinsic and sustained attention shared a common right hemisphere frontal parietal network. A previous study found that alertness-related regions were right dorsolateral prefrontal cortex, middle frontal gyrus, parietal lobe, part of the temporal lobe using the independent component analysis method,^[11]^ and right occipital lobe, cerebellum, right frontal lobe, and temporal lobe using the alertness task.^[12]^ The thalamus is known to be crucial in the alerting network.^[40,41,46,47]^ Kuncheng-Li and his companies also found that thalamus were associated with cognitive function, especial attention.^[48]^ It is widely known that alerting is a part of attention including alertness, oriention, and executive. Therefore, we speculated that thalamus was associated with alerting function. This study demonstrated that alertness-related regions were right superior frontal cortex, right precuneus, right middle temporal gyrus, right middle frontal gyrus, and right inferior temporal gyrus. Although the findings of the present study were quite consistent with those of the aforementioned studies, the small inconsistencies might have resulted from the heterogeneity of study samples and different research methods used. Another possible reason is that the brain network is dynamic, and the dysfunction of one brain region may contribute to the dysfunction of another brain region from the brain network perspective. All these results were combined with the findings of the present study, and it was postulated that an alertness-related network was involved in the aforementioned regions. Furthermore, FC significantly decreased in the right IPL, amygdala, and insula in the patient group compared with that in the control group. The facts that IPL comprises the parahippocampal gyrus, temporal lobe, and angular gyrus, and that the right IPL is crucial in the frontal parietal network, sustaining attention, alertness, and task switching are universally accepted. The temporal lobe is connected to the parietal lobe through the superior longitudinal fasciculus. Epileptic discharges from the right temporal lobe can damage these regions, leading to the decreased FC of related regions and alertness alterations in patients. A previous study found that some clinical characteristics were correlated with zFC in alertness-related regions.^[11]^ Therefore, a lack of correlation between these factors was somewhat unexpected. However, recent studies reported no correlation between these clinical characteristics and zFC.^[49]^ The reasons are as follows: First, the brain network is robust and dynamic, and the problem in one region may manifest itself differently in another region. Second, how these regions interact is still unclear.

This novel study found that FA values between the right insula and right superior temporal pole, right parahippocampal gyrus and right amygdala, right parahippocampal gyrus and right superior temporal pole, and right superior temporal gyrus and right superior temporal pole were lower in the patient group than in the control group. Moreover, significant negative correlations were found between abnormal FA values (between the right insula and the right superior temporal pole) and illness duration in the patients. Olson et al^[50]^ found that amygdala and temporal pole were associated with auditory and visual processing regions. Moreover, attention was verified to be associated with visual and auditory tasks. Moreover, alerting is known to be a part of attention, and attention comprises alerting, orienting, and executive.^[8]^ Recently, some researchers found that the 3 subnetworks were interconnected.^[13]^ In line with these studies, the lower FA values in the white matter of right parahippocampal gyrus, amygdala, superior temporal pole, and superior temporal gyrus disrupt communications between alertness-related regions. Moreover, an autopsy found that amygdala and superior temporal pole were obviously damaged in patients with epilepsy.^[51]^ Therefore, the results of the present study suggested that the white matter lesions in these regions might contribute to the pathophysiology of rTLE. Moreover, no correlation was found between the decreased FC regions and regions of lower FA values in alertness-related regions. Previous studies also had similar findings. For example, Honey et al^[52]^ found that the stronger the structural connectivity, the stronger the FC of these regions. However, this is not always true. Greicius et al^[53]^ found that the regions exhibiting FC had no direct white matter connectivity. Uddin et al^[54]^ found that a patient still had FC in the cerebral hemispheres after cutting off the former union. Therefore, the result was not surprising. Two possible explanations for the present results are as follows: First, the brain network is robust, and the function of alerting network is not affected if some regions are damaged; impaired white matter also has a compensation mechanism in patients. Second, a third subregion connecting these regions may exist. Furthermore, how white matter interacts with alerting function is not yet defined.

Repeated seizure activity could increase excitotoxicity.^[55]^ A recent study verified that the illness duration was correlated with lower FA values in certain regions in patients with TLE.^[56]^ As hypothesized, a significant negative correlation was found between abnormal FA values (right insula and right superior temporal pole) and illness duration in the patients. Therefore, it was speculated that illness duration might be a risk factor causing white matter impairment.

The present study had several limitations. First, data during human temporal lobe seizures were not obtained; hence, EEG was used. Second, the patients used various drugs for a long time, which might also have affected the results. Third, this study focused on the alertness network. Understanding the neurophysiological abnormalities of the alertness network in rTLE would be helpful. For the same reason, some meaningful findings from other brain regions besides the alertness network may be excluded. In addition, many evidences have demonstrated that there existed some morphological difference between Chinese population and others. Thus, it is also a limitation that the Chinese brain atlas were not used in present study.^[57,58]^ Finally, the effects of physiological noise at rest, such as cardiac and respiratory rhythm using a 2-s repetition time, could not be completely eliminated.

## 5 Conclusions

Despite several limitations, the present study examined the white matter lesions and decreased FC in alertness-related brain regions within rTLE. The findings suggested that the right IPL, amygdala, and insula might be important in alerting. This study provided an additional view for understanding the neurobiology of rTLE.

## Acknowledgments

The authors thank all individuals who served as research participants. They also appreciate anonymous reviewers for their suggestions and comments. Finally, the authors acknowledge the support granted from the Natural Science Foundation of China (Grant no. 81360202 and Grant no. 81560223).

## Author contributions

**Conceptualization:** Y. Li, Y. Gao.

**Data curation:** D. Guo, J. Zheng, M. Wang, W. Ye, X. Cui, Y. Li, Y. Gao.

**Formal analysis:** D. Guo, X. Cui, Y. Li, Y. Gao.

**Funding acquisition:** D. Guo, M. Wang.

**Investigation:** J. Zheng, W. Ye, X. Cui.

**Methodology:** D. Guo, W. Ye, Y. Li.

**Project administration:** J. Zheng.

**Resources:** X. Cui, Y. Li, Y. Gao.

**Software:** M. Wang, W. Ye, X. Cui.

**Supervision:** Y. Gao.

**Validation:** J. Zheng, W. Ye.

**Visualization:** M. Wang.

**Writing – original draft:** Y. Gao.

**Writing – review & editing:** D. Guo, J. Zheng, M. Wang, W. Ye, X. Cui, Y. Li.
